# Relationship between glucocorticoids and viral load during the Omicron wave in mainland China

**DOI:** 10.1186/s12985-023-02235-4

**Published:** 2023-11-22

**Authors:** Guangxu Bai, Yan Li, Yang Liu, Xinming Wang, Xuezhong Yu, Lili Ren, Jun Xu

**Affiliations:** 1 Emergency Department, State Key Laboratory of Complex Severe and Rare Diseases, Peking Union Medical College Hospital, Chinese Academy of Medical Science and Peking Union Medical College, Beijing, 100730 China; 2 Department of Clinical Laboratory, Peking Union Medical College, Peking Union Medical College Hospital, Chinese Academy of Medical Science, Beijing, 100730 China; 3 NHC Key Laboratory of Systems Biology of Pathogens and Christophe Mérieux Laboratory, Institute of Pathogen Biology, Chinese Academy of Medical Sciences & Peking Union Medical College, Beijing, 100730 China; 4 Key Laboratory of Respiratory Disease Pathogenomics, Chinese Academy of Medical Sciences, Peking Union Medical College, Beijing, China

**Keywords:** COVID-19, Glucocorticoids, Viral load, Cytokines, Viral clearance

## Abstract

**Background:**

Coronavirus disease 19 (COVID-19) is a major public health problem that cannot be ignored. As a widely used drug in the treatment of COVID-19, whether glucocorticoids may accelerate the clearance of COVID-19 is still not clear, and the glucocorticoids may improve the prognosis of patients is also controversial. Therefore, to explore the relationship between COVID-19 viral load and the use of glucocorticoids we designed this study.

**Methods:**

Patients with COVID-19 infection who were admitted to the emergency department of Peking Union Medical College Hospital from the end of 2022 to early 2023 were enrolled in this study. Characteristics of baseline, clinical and laboratory evaluation especially immunological indicator and daily viral load were carefully collected. Kolmogorov–Smirnov test, Student’s t test, Mann–Whitney U test and proportional-hazards model (Cox model) were chosen as appropriate for comparison of variables.

**Results:**

By comparing the daily COVID-19 viral load and prognosis of patients with and without glucocorticoid therapy, we found that glucocorticoids did not statistically enhance the clearance or replication of COVID-19, nor did it change the 28-days and in-hospital mortality. However, glucocorticoid therapy may be a favorable factor for COVID-19 negative conversion in Cox model. The inflammatory factors in patients with glucocorticoid therapy were significantly decreased.

**Conclusions:**

We believe that the real effect of glucocorticoids may be to improve the destruction of host immune system caused by inflammatory storm through host immune regulation and then achieve the improvement of clinical symptoms.

## Introduction

Since 2019, coronavirus disease 19 (COVID-19) has spread widely in hundreds of countries and regions worldwide, resulting in a cumulative total of 768 million confirmed cases and 6.94 million deaths as of June 2023 (https://www.who.int/data). Despite the widespread availability of vaccines and antivirals [1] and numerous trials of new or reactivating drugs [2–4], therapeutic agents that reduce mortality from COVID-19 remain unavailable. During the late 2022 pandemic caused by the BF.7 and BA.5.2 strains, China experienced very serious challenges. Therefore, identifying better treatment and prevention solutions for COVID-19 infection has been an urgent problem to be solved.

Glucocorticoids, especially the classic drug dexamethasone, are widely used in the treatment of COVID-9 infection [5]. Dexamethasone’s powerful and sustained anti-inflammatory effect has been reaffirmed in numerous studies [6, 7]. Based on the results of the RECOVERY study [8], dexamethasone effectively decreases 28-day mortality and the risk of mechanical ventilation in hospitalized patients with COVID-19. Moreover, the COVID-19 treatment guideline [9] strongly recommends the early and combination use of corticosteroids to improve pulmonary symptoms and prognosis in severe or critical patients. However, given the powerful immunosuppressive effect of glucocorticoids, viral replication may be aggravated in previous viral infections such as hepatitis B infection [10]. Recent studies of influenza infection, Middle East Respiratory Syndrome, and other coronavirus infections [11–13] have further indicated that the use of glucocorticoids may be associated with delayed viral clearance and longer hospital stays. Therefore, we designed this study to analyze the relationship between glucocorticoids and viral load in COVID-19 infection by collecting the data of patients diagnosed with COVID-19 infection when the COVID-19 pandemic occurred in mainland China in early 2023.

## Methods

### Study population and design

Patients who were diagnosed with COVID-19 infection and admitted to the emergency department of Peking Union Medical College Hospital from December 2022 to January 2023 participated in this prospective study. All of the patients signed informed consent forms and the study was approved by the hospital’s ethics committee. This study was approved by the Ethics Committee of PUMCH and registered at chictr.org.cn (identifier ChiCTR2000030349). Inclusion criteria were (1) duration of hospitalization ≥ 48 h and (2) confirmed severe and critical COVID-19 infections. The exclusion criteria were (1) mild and moderate patients with confirmed COVID-19 infection and (2) all patients who used antiviral drugs during their illness, including before the visit to the doctor. In accordance with the diagnosis and treatment protocol for COVID-19 (9th & 10th edition) established by the National Health Commission of the People’s Republic of China, COVID-19 infection is defined as having (1) clinical manifestations related to COVID-19 infection and (2) one or more of the following etiological and serological test results: (a) positive COVID-19 nucleic acid test, (b) positive COVID-19 antigen, (c) positive COVID-19 isolation and culture, and (d) a level of novel coronavirus-specific immunoglobulin G antibody in the convalescent phase that is equal or greater than four times that in the acute phase. The clinical classification was defined as follows. “Mild” referred to the mild manifestations of the main clinical symptoms of upper respiratory tract infection, such as dry throat, sore throat, cough, and fever. “Moderate” referred to persistent high fever for > 3 days or cough, or both, shortness of breath with a respiratory rate (RR) < 30 times/min and oxygen saturation > 93% when breathing air at rest, and imaging that showed the characteristic manifestations of COVID-19 pneumonia. A classification of “severe” was used for adults who met any of the following criteria that could not be explained by anything other than COVID-19 infection: (a) shortness of breath (i.e., RR ≥ 30 times/min), (b) oxygen saturation < 93% when breathing air at rest, (c) arterial partial oxygen pressure/oxygen absorption concentration ≤ 300 mmHg (1 mm Hg = 0.133 kPa), and (d) progressively worsening clinical symptoms and lung imaging that showed that the lesion had progressed significantly (i.e., > 50%) within 24 to 48 h. Critical patients were defined as those who has one of the following conditions occurs: (1) respiratory failure requiring mechanical ventilation; (2) shock; (3) complicated with other organ failure requiring ICU care.

All of the enrolled patients were labeled as being in early (0–7 days), convalescent (8–14 days), and advanced (> 14 days) stages based on their symptoms (fever, sore throat, cough, and dyspnea) or the time of their first positive COVID-19 nucleic acid test. Since the criteria for glucocorticoids were severe and critical patients with progressive deterioration of oxygenation indexes, rapid imaging progress, and excessive activation of the body’s inflammatory response according to the diagnosis and treatment protocol for COVID-19 (9th edition) established by the National Health Commission of the People’s Republic of China, after enrollment, at least two experienced clinicians assessed the need for glucocorticoids, which were administered orally at a dose of 5 mg dexamethasone daily.

### Clinical and laboratory evaluation

All of the enrolled patients underwent a comprehensive clinical and laboratory evaluation on the day of hospitalization. The information recorded included age, sex, underlying diseases, important biochemical indicators of infection (e.g., procalcitonin, creatinine, albumin, bilirubin, and coagulation), Acute Physiology And Chronic Health Evaluation II (Apache-II) [14] and Sequential Organ Failure Assessment (SOFA) [15] scores, and a record of any life-sustaining treatments that lasted ≥ 24 h (e.g., mechanical ventilation, non-invasive positive pressure ventilation, high-flow nasal catheter, Venturi mask and extracorporeal membrane oxygenation, vasopressor drugs, or renal replacement therapy).

### Immunological laboratory examination

Serum was obtained from all of the patients to test immune parameters, including white blood cell count, complement, immunoglobulin, inflammatory factors, T cell subsets, and ferritin. In brief, the detection of T cell subsets began with the isolation of peripheral blood mononuclear cells, which were then stained with different combinations of fluorescent monoclonal antibodies. Finally, T cells (CD3^+^), CD4^+^ T cell subsets (CD4^+^CD3^+^ and CD28^+^CD4^+^), CD8^+^ T cell subsets (CD8^+^CD3^+^, CD28^+^CD8^+^), B cells (CD19^+^), and NK cells (CD3^−^CD16^+^CD56^+^) were detected using flow cytometry.

### RNA extraction and reverse transcription-polymerase chain reaction (RT-PCR) detection of COVID-19 viral load in throat swabs

Throat swabs were taken daily from all of the enrolled patients for COVID-19 viral load testing. The patients were instructed to rinse their mouths with water and a swab was inserted in the mouth and passed over the base of the tongue. Both sides of the pharyngeal tonsils were first swabbed back and forth at least three times and then we’re swabbed over and down the back wall of the pharynx at least three times. The swab was withdrawn and placed into a collection tube. All of the swabs were collected and soaked in 1,000 μl phosphate buffer saline. After 30 s of shaking, 400 μl of the sample was removed for nucleic acid extraction with the SY619 nucleic acid extraction kit (Suzhou Xinbo Biotechnology Co. Ltd., Suzhou, China) in accordance with the manufacturer’s instructions. The number of viral copies was measured using RT-PCR using primers and probes that target the N gene of the severe acute respiratory syndrome coronavirus 2 (SARS-Cov-2). The reference standard was diluted tenfold from 1 × 10^8^ copies to 1 × 10^9^ copies. The PCR amplification cycle was 15 min at 50 °C, 3 min at 95 °C, 15 s at 95 °C, 45 s at 60 °C and then plate-read for 50 cycles. The amplification process, fluorescence signal detection, data storage, and analysis were all completed by quantitative fluorescence PCR and its software Bio-Rad CFX Manager (Bio-Rad Laboratories Co. Ltd., California, USA). The number of copies of the virus was calculated according to the standard curve and then converted to log 10 for statistical analysis. COVID-19 negative conversion was defined as two consecutive cycle threshold (CT) values of SARS-CoV-2 nucleic acid *N* gene and *ORF* gene ≥ 35.

### Statistical analysis

All analyses were performed using SPSS for Windows version 24.0 (IBM Corp., Armonk, NY, USA). The Kolmogorov–Smirnov test, Student’s t-test, and Mann–Whitney U test were used to examine the cumulative distribution functions and analyze the normally distributed continuous and nonparametric variables. The chi-square or Fisher exact test was chosen as appropriate for the comparison of categorical variables. P values associated with “equal variances not assumed” were reported for variables that violated the homogeneity of variance assumption. All tests performed were two-tailed, and P < 0.05 was considered statistically significant.

## Results

### Patient characteristics

**Fig. 1:**
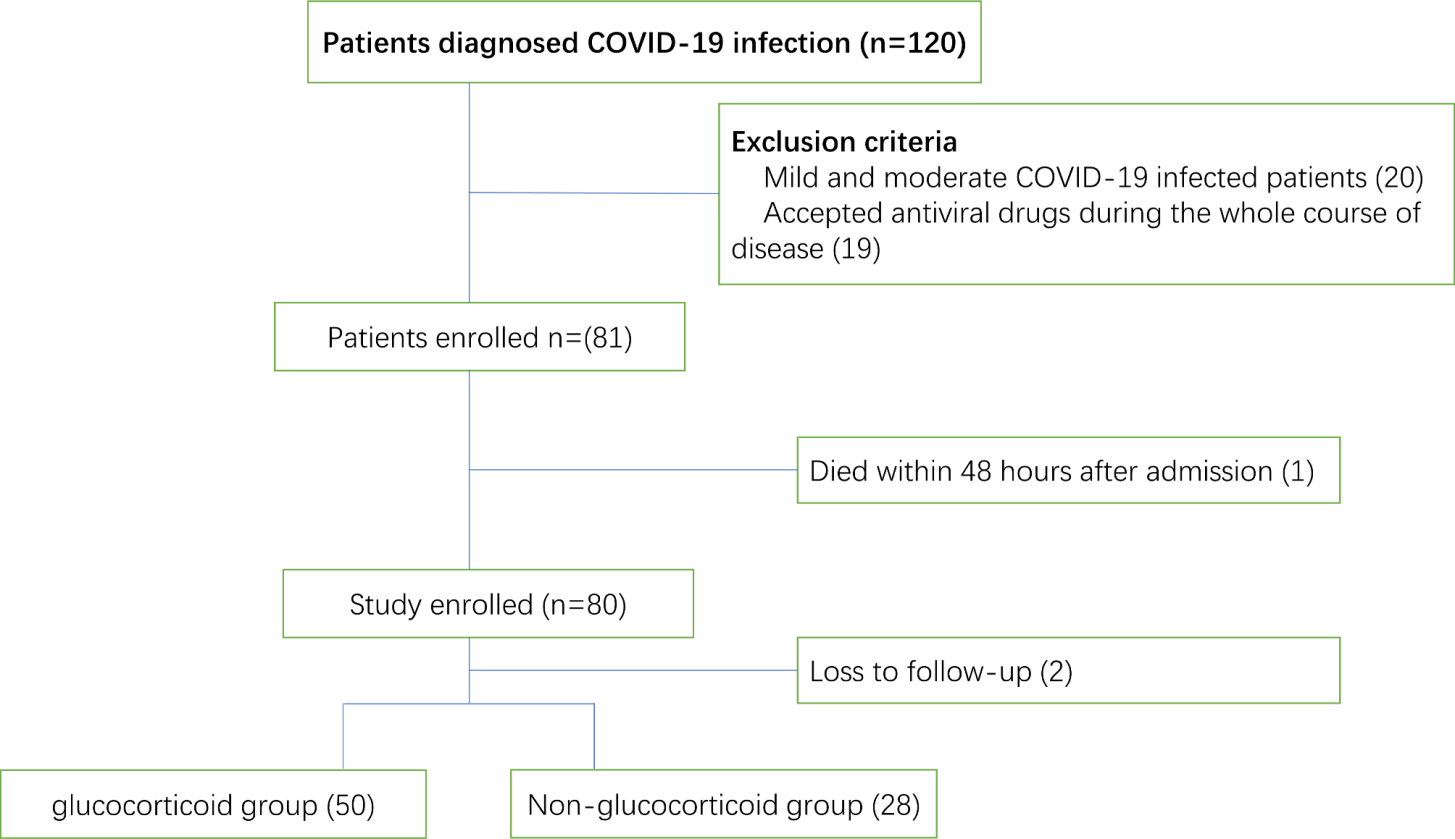
Flowchart for the selection of the study population

A total of 120 patients with COVID-19 infection caused by Omicron who were admitted to the emergency department were included in the study. Thirty-nine patients were excluded as they had taking antiviral medication before admitting to emergency, one patient died within 48 h of inclusion, and two patients were lost to follow-up. The remaining 78 patients were included in the study (Fig. 1), with 50 patients in the glucocorticoid group and 28 patients in the non-glucocorticoid group. The Omicron lineages were inferred by Nextclade from the nearest neighbor in the reference tree of the Pango lineage database. The majority of Omicron strains were BF 7.1.4 and its polytypic subclades BF 7.1.4.2 and BF 7.1.4.6. No statistically significant differences in baseline and clinical characteristics were observed between the two groups, including differences in sex, age, time of vaccination, stage of COVID-19 infection, underlying disease, SOFA score, APACHE-II score, Murray score, clinical pulmonary infection score, life-sustaining treatments, biochemical, immune parameters and coagulation indicators, or whether other etiological infections were combined (Tables 1 and 2).

**Table 1:** Baseline characteristics of the study population

Variables	Covid-19 infected (n = 78)	Glucocorticoid used (n = 50)	Non-Glucocorticoid used (n = 28)	*P* value
Age (years)	74.5(17.5)	76 (17)	71.5 (22)	0.889
Sex (male: female)	49:29	33:17	16:12	0.437
Vaccination (vaccinated: unvaccinated)	42:36	26:24	16:12	0.093
Phase of onset (early: convalescence: late)	22:38:18	14:24:12	8:14:6	0.966
**Underlying disease (n (%))**				
COPD	10 (12.8)	8 (16)	2 (7.1)	0.262
Heart failure	5 (6.4)	2 (4)	3 (10.7)	0.245
Diabetic mellitus	26 (33.3)	19 (38)	7 (25)	0.243
Liver Cirrhosis	4 (5.12)	3(6)	1 (3.6)	0.641
Tumor	8 (10.3)	7 (14)	1 (3.6)	0.145
Chronic renal failure	6 (7.7)	5(10)	1 (3.6)	0.307
APACHE II score	11 (5)	11 (4)	15(7)	0.174
SOFA score	1 (1)	1 (1)	1 (2)	0.156

Data are number of patients (%) or median and interquartile range (median [IQR]). APACHE-II: Acute Physiology And Chronic Health Evaluation II; COPD: chronic obstructive pulmonary disease; SOFA: Sequential Organ Failure Assessment

**Table 2:** The clinical characteristics of patients infected with COVID-19

Variables	Covid-19 infected (n = 78)	Glucocorticoid used (n = 50)	Non-Glucocorticoid used (n = 28)	*P* value
**Life-sustaining treatments (n (%))**				
Mechanical ventilation	7(9)	4 (8)	3 (10.7)	0.687
NIPPV	3(3.8)	2(4)	1 (3.6)	0.925
HFNC	28(35.9)	20(40)	8(28.6)	0.313
Venturi Mask	46(59)	32(64)	14(50)	0.228
ECMO	1(1.3)	1(2)	0	0.451
Need for vasopressor	7 (9)	3 (6)	4 (14.3)	0.219
Need for RRT	1 (1.3)	1(2)	0	0.451
**Other Pathogens (n (%))**				
Bacteria	16 (20.5)	12 (24)	4 (14.3)	0.308
Fungal	12 (15.4)	8 (16)	4 (14.3)	0.84
**Biochemical parameters at Emergency admission (median (IQR))**				
Albumin (g/L)	33.5 (7)	34.5(6)	33(7)	0.607
Serum Creatinine (μmol/L)	86 (41)	89(60)	80.5(42)	0.185
Total Bilirubin (μmol/L)	19.9 (31.2)	19.6(37)	20.9(25.1)	0.149
D-dimer (mg/L)	1.88 (8.68)	1.88(10.47)	1.91(8.02)	0.447
**Infection marker at Emergency admission (median (IQR))**				
Procalcitonin (ng/mL)	0.15 (0.58)	0.16 (0.5)	0.11(0.68)	0.757
CPIS	6 (2)	6 (2)	6 (2)	0.34
Murray Score	2(2.67)	2(1.17)	1.75(2.17)	0.319
**Outcome**				
Hospital stay (days) (median(IQR))	13.5(10)	13.5(10)	12.5 (10)	0.562
in-Hospital mortality (n(%))	14 (17.9)	10 (20)	3 (10.7)	0.291
28 days mortality (n(%))	12 (15.4)	9 (18)	3 (10.7)	0.392

Data are number of patients (%) or median and interquartile range (median [IQR]); NIPPV: nasal intermittent positive pressure ventilation; HFNC: high flow nasal cannula; ECMO: extracorporeal membrane oxygenation; RRT: renal replace treatment; CPIS: Clinical pulmonary infection scores

### Comparison of immune parameters in patients who took or did not take glucocorticoid therapy

Table 3 shows the comparative analysis of some commonly used clinical immune indicators (leukocytes, complement, immunoglobulin, inflammatory factors, T cell subsets, and ferritin) between the two groups at admission; no significant statistical difference was observed.

**Table 3:** Immune parameters of patients in the glucocorticoid and non-glucocorticoid groups

Variables	Covid-19 infected (n = 78)	Glucocorticoid used (n = 50)	Non-Glucocorticoid used (n = 28)	*P* value
WBC (10^9^/L)	8.36 (5.6)	7.39 (6.37)	9.11 (4.57)	0.834
NG (10^9^/L)	6.86 (5.24)	4.98 (4.49)	8.1 (5.7)	0.081
NK cells (/mm^3^)	60 (79)	52 (90)	71 (76)	0.548
B cells (/mm^3^)	87 (85)	71 (69)	99 (102)	0.091
**Lymphocyte (cells/mm**^**3**^)	565 (723)	515 (430)	667 (883)	0.144
CD4^+^T	260 (285)	187 (236)	301 (320)	0.188
CD28^+^CD4^+^T	226 (297)	158 (234)	260 (332)	0.431
MeT4	155 (192)	98 (148)	198 (205)	0.741
RAT4	73 (149)	53 (125)	73 (199)	0.229
NaT4	50 (105)	50(121)	50 (95)	0.111
CD8^+^T	138 (185)	113 (123)	176(236)	0.054
CD28^+^CD8^+^T	55 (62)	48 (66)	59 (75)	0.289
DRT8	60 (127)	51 (96)	68 (146)	0.122
CD38^+^T8	75(126)	68 (91)	96 (173)	0.824
**Complement factor (g/L)**				
C3	0.94 (0.43)	0.96 (0.42)	0.91 (0.58)	0.471
C4	0.19 (0.11)	0.19 (0.10)	0.19 (0.10)	0.448
**Immunoglobulin (g/L)**				
IgA	2.27 (1.8)	2.26 (1.74)	2.42 (2.18)	0.358
IgG	11.38 (7.8)	11.09 (8.2)	11.46 (7.3)	0.527
IgM	0.73 (0.76)	0.62 (0.39)	0.85 (0.82)	0.492
**Cytokines (g/L)**				
IL-6	36.5 (66.23)	34.4 (68.8)	36.5 (74.93)	0.833
IL-8	145 (460)	212(429.25)	51(592.5)	0.166
IL-10	7.5 (9.25)	6.75(4.03)	8.05(9.15)	0.464
TNF-α	15.25 (12.1)	15.4(12.05)	14.95(14.55)	0.384
**Serum Ferritin (μg/L)**	433 (627.75)	505 (573)	392.5 (654)	0.098

Data are number of patients (%) or median and interquartile range (median [IQR]). NG: neutrophile granulocyte; NK: natural killer cell; WBC: white blood cell count

### Correlation between COVID-19 viral load and disease severity

**Fig. 2:**
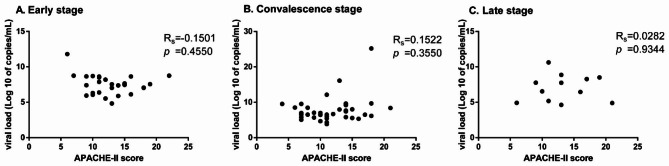
Correlation between COVID-19 viral load and APACHE-II score on the day of hospitalization in patients at each stage

In order to determine whether COVID-19 viral load may reflect the severity of the disease, we first conducted correlation analysis between the first-day viral load and APACHE-II score, a commonly used clinical score for evaluating the disease severity on the day of hospitalization in patients at each stage. Figure 2 shows that no significant statistical correlation between COVID-19 viral load and disease severity.

### Comparison of daily (day 1–9) virus load of patients infected with COVID-19 in different stages

**Fig. 3:**
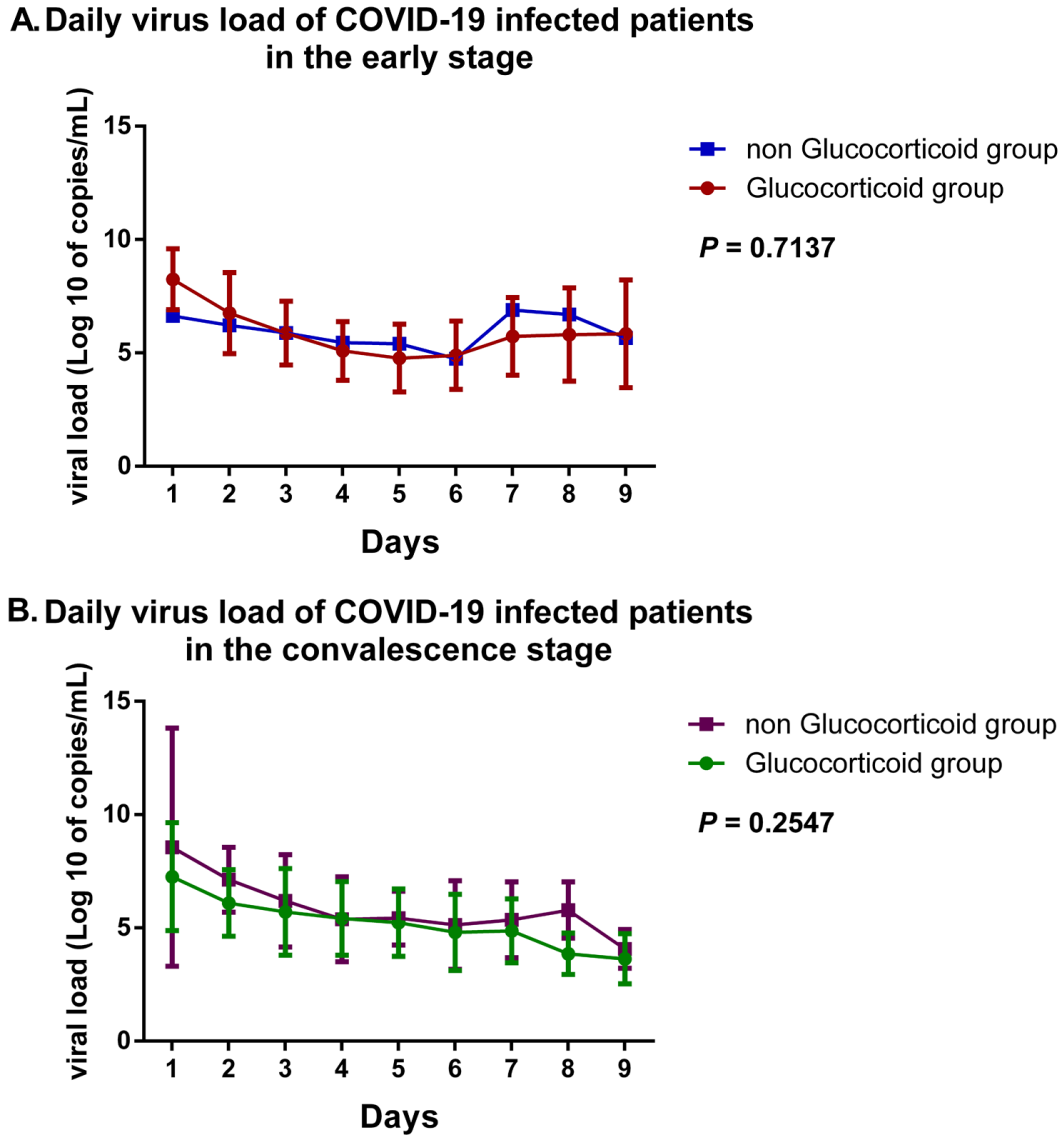
Comparison of daily (Day 1–9) virus load of patients infected with COVID-19 in different stages

**Fig. 4:**
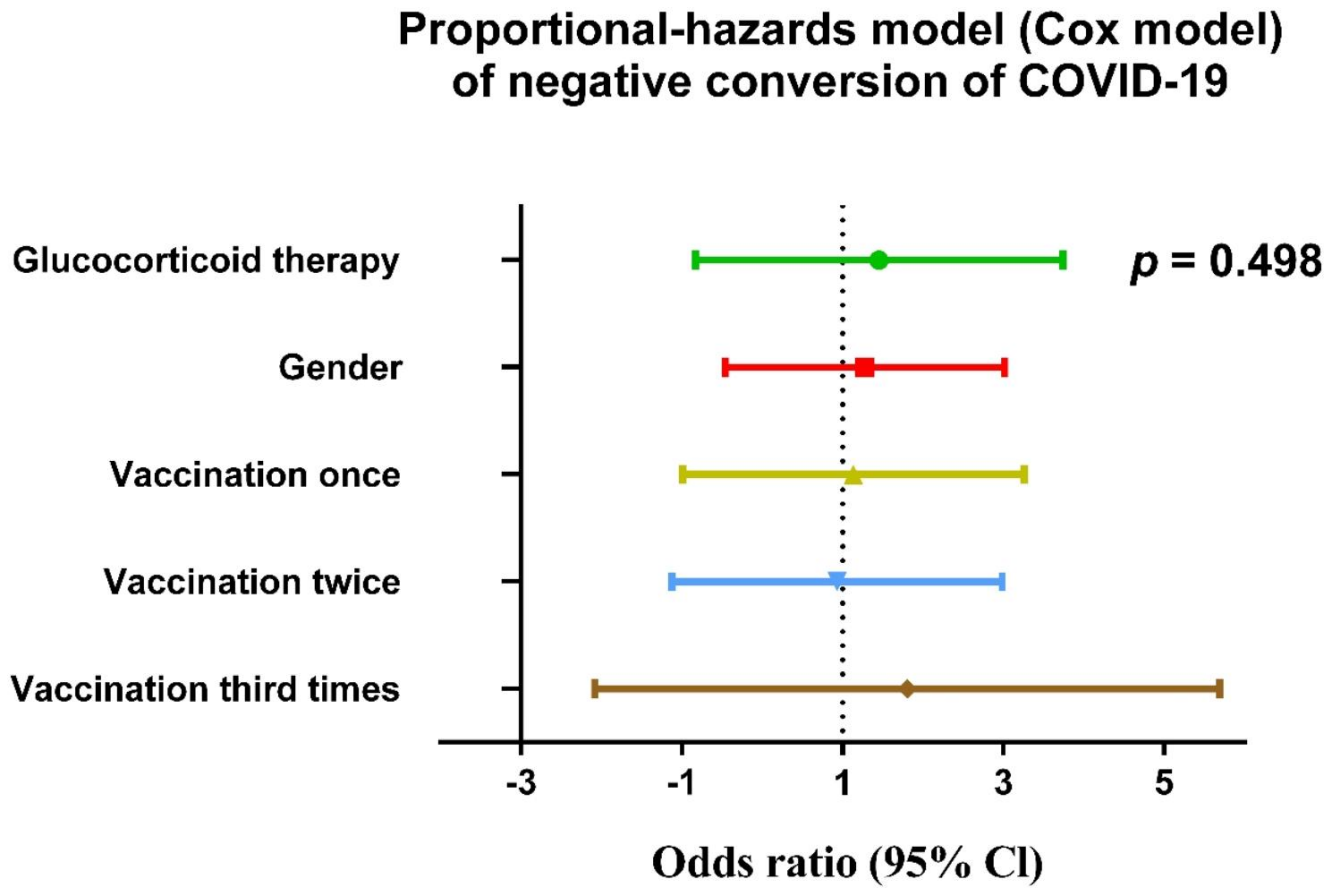
Forest plot of potential risk factors related to the negative conversion of COVID-19

To visually analyze the effect of glucocorticoid use on COVID-19 viral load, we performed daily (from Day 1 to Day 9) comparisons of viral load in different stages patients who took or did not take glucocorticoid therapy. In view of the late stage of the viral infection and the severity of the disease, all the patients in the late stage in this study accepted glucocorticoid therapy. Figure 3 shows a lack of statistically significant differences in daily viral load between patients in early and convalescent stages who accepted glucocorticoid therapy and those who did not. However, in the proportional-hazards model (Cox model) of negative conversion of viral load, we found that treatment with glucocorticoids may promote the negative conversion of COVID-19, although this effect was not statistically significant as shown in Fig. 4.

### Comparison of inflammatory factors in patients infected with COVID-19 who took or did not take glucocorticoid therapy

**Fig. 5:**
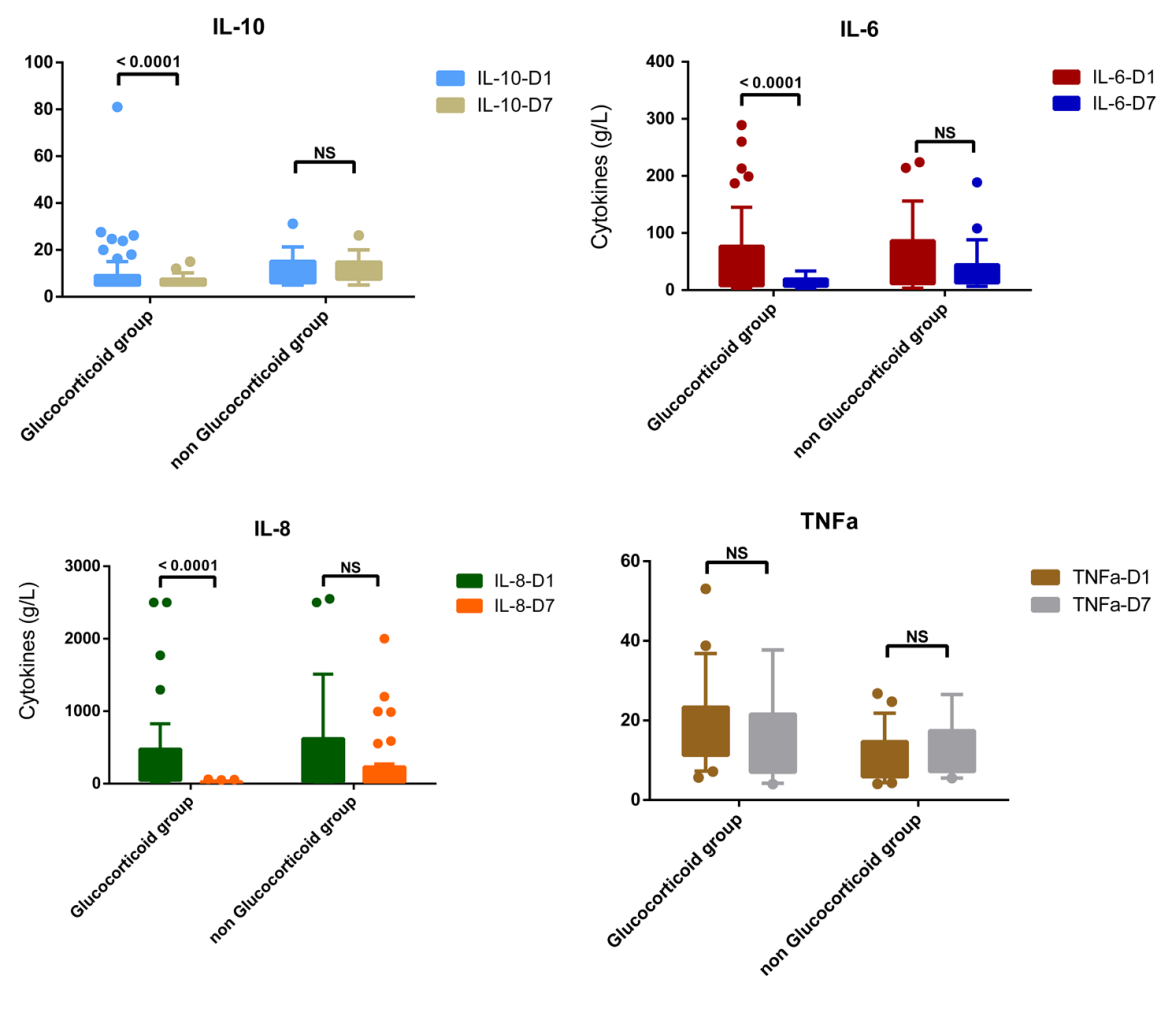
Comparison of inflammatory factors in patients with COVID-19 infection who took or did not take glucocorticoids

Since cytokines may reflect the intensity of the host inflammatory response, in order to explore the effect of corticosteroids on the host inflammatory response in the course of the disease, we compared the levels of cytokines in patients with and without glucocorticoid therapy. Figure 5 shows the changes in inflammatory factors in patients who took or did not take glucocorticoid therapy. Compared with patients who did not take glucocorticoids, the levels of inflammatory factors IL-6, IL-8, and IL-10 in patients who used glucocorticoids were significantly reduced. However, no statistically significant difference in TNF-a levels was observed between the groups.

## Discussion

To the best of our knowledge, this is the first clinical study to analyze the relationship between glucocorticoids therapy and Omicron viral load and negative conversion in COVID-19 infection. Even today, COVID-19 infection remains a considerable threat to human health. Although the global campaign against COVID-19 has achieved some phased results, the extremely strong mutation capacity and unpredictable virulence of COVID-19 have always been an urgent problem that has confronted humankind.

Glucocorticoids have a strong anti-inflammatory effect. Their powerful anti-inflammatory activity is attributed to their steroid receptor glucocorticoid receptor, which inhibits significant pro-inflammatory gene expression through signal transduction and plays a dual role in regulating the immune response [16]. Therefore, whether in the treatment of bacterial or viral infection, glucocorticoids are widely used and are often used as impact and salvage therapy, especially in viral pneumonia [5].

However, the dosage and timing of glucocorticoids in the treatment of viral pneumonia have been controversial [17]. During the 2003 severe acute respiratory syndrome (SARS) epidemic, observational studies from the Chinese mainland and Hong Kong [18, 19] have reported the beneficial effects of glucocorticoid therapy. Another study [20] has shown that glucocorticoids are associated with increased illness in patients. In 2009, when severe pneumonia was caused by the outbreak of the influenza A (H1N1) virus, Han et al. [21] showed that early treatment with fast-acting corticosteroids in patients with severe pneumonia did not improve inflammatory indicators such as C-reactive protein (CRP) and erythrocyte sedimentation rate (ESR). Nevertheless, the authors observed that a comparison of imaging, clinical respiratory support conditions, and prognostic indicators in patients treated with and without corticosteroid therapy demonstrated that glucocorticoids may not only prevent clinical and radiographic deterioration but also the onset and progression of acute respiratory distress syndrome. Other studies [22, 23] have shown that the early use of glucocorticoids in pH1N1 infection may be associated with an increased risk of subsequent critical illness or death. Therefore, the WHO Guidelines for Pharmacological Management of Pandemic Influenza A(H1N1) 2009 and Other Influenza Viruses issued by the World Health Organization [24] recommends against the use of corticosteroids in the treatment of patients with pneumonia caused by H1N1 influenza pandemics.

In the early stage of the COVID-19 pandemic, given the lack of vaccines and effective antiviral drugs, the mortality of patients with COVID-19 was greater than 30% despite aggressive respiratory support including mechanical ventilation and extracorporeal membrane oxygenation (ECMO) [25]. In such a dilemma, glucocorticoid therapy seems to be one of the few options for clinicians. The two large randomized controlled trials of glucocorticoid therapy have similarly conflicting conclusions [8, 26].

The main concern about the use of glucocorticoids for the treatment of COVID-19 infection is that the powerful immunosuppressive effects of these agents may worsen viral replication. A previous study [27] has shown that although dexamethasone limits the production and destruction of cytokines, it also inhibits the protective function of T cells and prevents B cells from producing antibodies, potentially leading to an increase in plasma viral load that persists after patients who are infected with SARS-Cov-2 survive.

As one of the most important and intuitive parameters after viral infection, viral load can be used to evaluate the effectiveness of COVID-19 treatment. Therefore, in this study, we prospectively compared the number of copies of SARS-CoV-2 in patients who took or did not take glucocorticoid therapy to assess the effect of glucocorticoids on viral load and negative conversion in the host. However, in viral infections, the host viral load may not parallel the severity of the disease [28]. To explore whether COVID-19 viral load may reflect the severity of the disease, we first conducted a correlation analysis between the first-day viral load and APACHE-II score. We found that the viral load of the host did not reflect disease severity (Fig. 2).

We then compared the daily viral load dynamics of the two groups separately according to the onset of symptoms and did not observe a statistically significant effect of glucocorticoids therapy on patients’ COVID-19 viral load (Fig. 3). Nevertheless, the treatment with glucocorticoids may promote the negative conversion of COVID-19 as shown in a Cox model (Fig. 4). In order to find potential influencing factors, we compared the changes of inflammatory indicators between the two groups,; we found patients who received glucocorticoid therapy had a significantly lower level of inflammatory factors than patients who did not receive glucocorticoid therapy (Fig. 5). This result was consistent with Kino et al. [29]. We believe that glucocorticoids play a more immunomodulatory role, which may avoid further immune imbalance and promote virus clearance by reducing the degree of host inflammatory response. Similarly with CODEX [6], another randomized clinical trial published in the *Journal of the American Medical Association* recently, we also found that glucocorticoid treatment had no significant effect on the outcomes such as the length of hospital stay, the 28-days mortality of COVID-19 infection.

Our study had some limitations. First, this was a single-center prospective study with a small sample size. Multi-center randomized controlled trials are planned to further analyze the role of glucocorticoids in Omicron infection. Second, we recorded data at a single time point and did not conduct dynamic observation at multiple time nodes. To address this, we plan to conduct multiple data collections at different times to support further analysis of the regulatory effect of glucocorticoids on host immunity in COVID-19 infection.

## Conclusion

The viral load on the day of admission could not reflect the severity of the disease due to individual differences in the strength of the baseline immune response of the host individuals. There was no significant difference in the dynamics of daily viral load and the outcome of viral negative conversion between the two groups of patients with or without glucocorticoid therapy. However, Cox regression showed that glucocorticoid therapy may be a favorable factor for COVID-19 negative conversion. By comparing the changes of inflammatory indicators of patients with COVID-19 who took or did not take glucocorticoid therapy, we believe that the real effect of glucocorticoids may be to improve the destruction of host immune system caused by inflammatory storm through host immune regulationand then achieve the improvement of clinical symptoms. Although our study has obtained a large number of negative results, we still believe that may help us to further explore COVID-19 infection from the perspective of immune regulation, better define the site and timing of glucocorticoid application, and promote a deeper understanding of the host immune response to COVID-19 infection, so as to find new treatment directions and targets.

## Data Availability

The data set supporting the results of this article is included within the article.
